# Carcinome épidermoïde du sein: à propos de 3cas et revue de la littérature

**Published:** 2012-06-21

**Authors:** Fatima Zohra Fdili Alaoui, Saad Benkirane, Hekmat Chaara, Hakima Bouguern, Moulay Abdilah Melhouf

**Affiliations:** 1 Service de Gynécologie-Obstétrique II, CHU Hassan II, Fès, Maroc

**Keywords:** Carcinome épidermoide, sein, diagnostic, traitement, pronostic

## Abstract

Les carcinomes épidermoides du sein sont rares. Ils sont d'origine métaplasiques. Leur histogénèse est controversée. La présentation clinique et mammographique n'est pas spécifique, l'aspect kystisé des lésions et la présence de nécrose sont recherchés à l’échographie mammaire. Le diagnostic est histologique. Ce cancer est réputé être peu lymphophile et non hormonodépendant. Le traitement rejoint celui des carcinomes infiltrants canalaires et repose sur la chirurgie, la radiothérapie et la chimiothérapie. Le pronostic est péjoratif. Nous rapportons trois cas de carcinome épidermoide du sein colligés au service de Gynécologie obstétrique II au CHU Hassan II de Fès et une revue de la littérature.

## Introduction

Les carcinomes épidermoïdes primitifs du sein sont des tumeurs rares qui représentent 0,1% à 2% des cancers mammaires. Ils font partie des carcinomes métaplasiques du sein, et sont d’étiopathogénie et de pronostic controversés [1]. Nous rapportons trois cas de carcinome épidermoïde du sein, à travers lesquels et à la lumière de la littérature, nous précisons quelques caractéristiques de cette forme particulière de cancer du sein.

## Patients et observations

### Observation 1

Madame LA, âgée de 32 ans, mère de 3 enfants, sans antécédent pathologique particulier présentant depuis un an un nodule du sein gauche ayant augmenté progressivement de volume jusqu’à l'occupation de la quasi-totalité du sein sans modifications cutanées ni écoulement mamelonnaire, ceci l'a motivé à consulté chez un gynécologue à titre externe, qui a trouvé une tuméfaction mammaire du sein gauche prenant presque la totalité du sein classée T3 N2Mx; une mammographie-échographie mammaire a objectivé un nodule tissulaire de 3 cm du quadrant supéro externe (QSE) du sein gauche d'allure suspecte, avec adénopathies homolatérales axillaires de 16, 13 et 12 mm. Une biopsie tumorale a été réalisée revenant en faveur d'un carcinome épidérmoïdegrade III SBR, récepteurs hormonaux négatifs, Hercept test positif de score 2. La patiente a bénéficié d'un bilan préchimiothérapique(échographie abdomino-pelvienne, radio pulmonaire et scintigraphie osseuse) revenu normal, puis a été adressée au service d'oncologie ou elle a reçu 6 cures d'anthracyclines (AC 60) et devant la non amélioration sur le plan clinique de la tuméfaction mammaire, elle a reçu 3 cures supplémentaires de taxanes puis a été adressée dans notre service pour prise en charge chirurgicale.

L'examen trouve une masse rétro aréolairedu sein gauche de 8/7cm adhérente aux 2 plans mal limitée dure irrégulière avec rétraction mamelonnaire; le sein droit, les aires ganglionnaires et les autres appareils étaient sans particularité. La patiente a donc bénéficié d'une mastectomie totale avec curage ganglionnaire. L'examen anatomopathologiaque est revenu en faveur d'un carcinome épidermoïde grade III SBR, de 2,5cm, présence d'une composante intracanalaire de haut grade massive et cribriforme, réponse thérapeutique grade C de Sataloffet, présence d'emboles vasculaires avec nécrose, 1ganglion lymphatique était envahi. La patiente a été re-adressée au service d'oncologie pour traitement adjuvant.

### Observation 2

**Figure 1:**
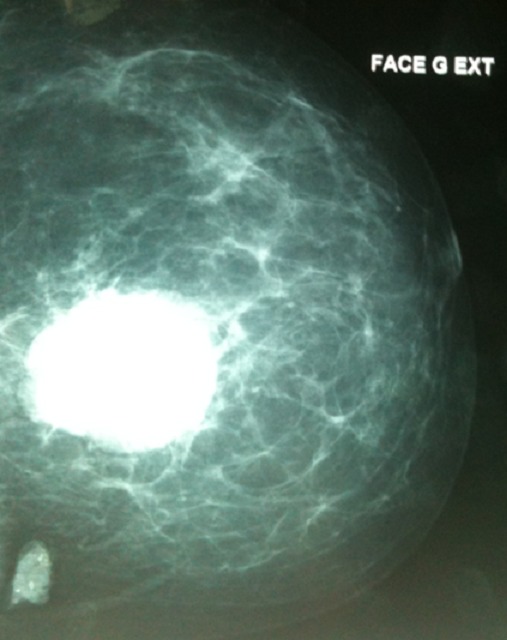
Mammographie de face de Madame GF montrant une opacité du sein gauche au niveau du quadrant supérointerne classée ACR5

**Figure 2:**
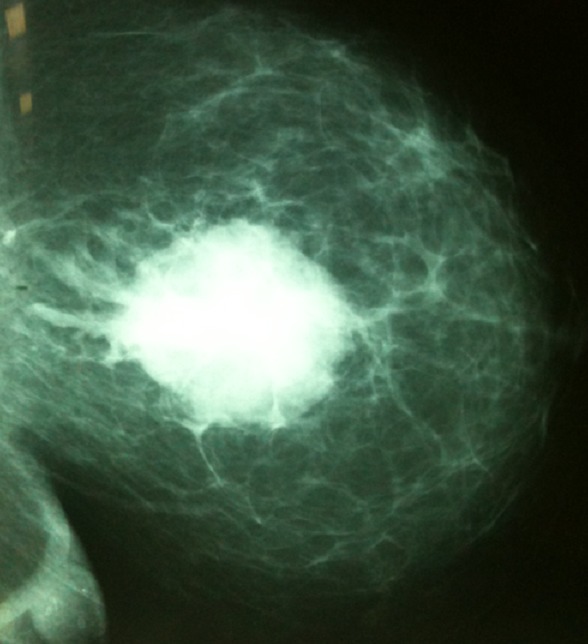
Mammographie de profil de Madame GF montrant une opacité du sein gauche au niveau du quadrant supérointerne classée ACR5

**Figure 3:**
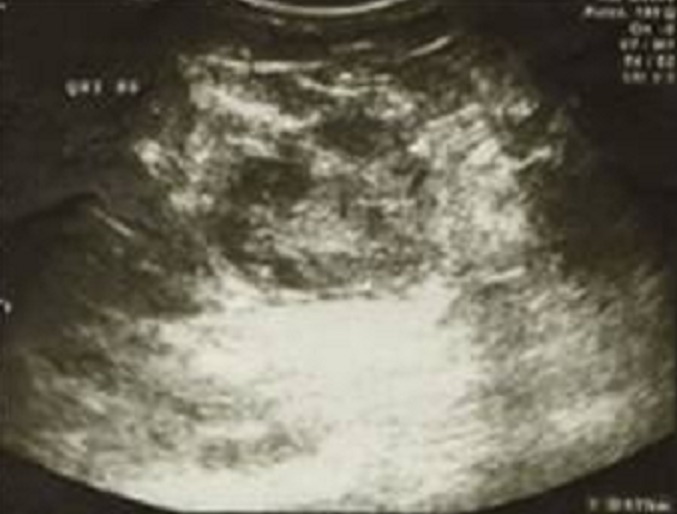
Échographique de Madame GF montrant une lésion tissulaire hétérogène (4,7cm) du quadrant supérointerne du sein gauche classée ACR5

Mme GF, âgée de 57ans, grande multipare, ménopausée,sans antécédent pathologique particulier, a consulté pour tuméfaction du sein gauche découverte à l'autopalpation ayant augmenté progressivement de volume, L'examen initial a objectivé une masse du quadrant supéro interne (QSI) de 9cm irrégulière dure mobile, classée cliniquement T3N0Mx. Une mammographie échographie mammaire a objectivé (Figure 1, Figure 2, Figure 3): une lésion tissulaire du QSI du sein gauche classée ACR 5 (4,7cm), une autre du même quadrant de 10mm avec adénopathies homolatérales infra centimétriques. Une biopsie a été réalisée et est revenue en faveur d'un carcinome épidermoïde. Récepteurs hormonaux négatifs, KI67: 90%, Hercept test négatif: score 0. Le bilan préchimiothérapique (échographie abdominopelvienne, radio thoracique, TDM thoraco abdomino pelvienne, et scintigraphie osseuse) était normal. La patiente a été prise en charge dans le service d'oncologie ou elle a reçu 6 cures de Cisplatine, Taxanes, la patiente nous a été adressée pour geste chirurgicale. L'examen a trouvé une masse du QSI du sein gauche (11/10cm) adhérente au plan superficielle, ulcérée par endroit. Le sein controlatéral, les aires ganglionnaires et le reste de l'examen étaient sans particularité. Une mastectomie totale avec curage ganglionnaire a confirmé la présence du carcinome épidermoïde (6,6cm) moyennement différencié et mature grade TCNB de Sataloff et grade 3 de chevallier sans envahissement ganglionnaire. La patiente a été ensuite réadressée au service d'oncologie pour traitement adjuvant.

### Observation 3

**Figure 4:**
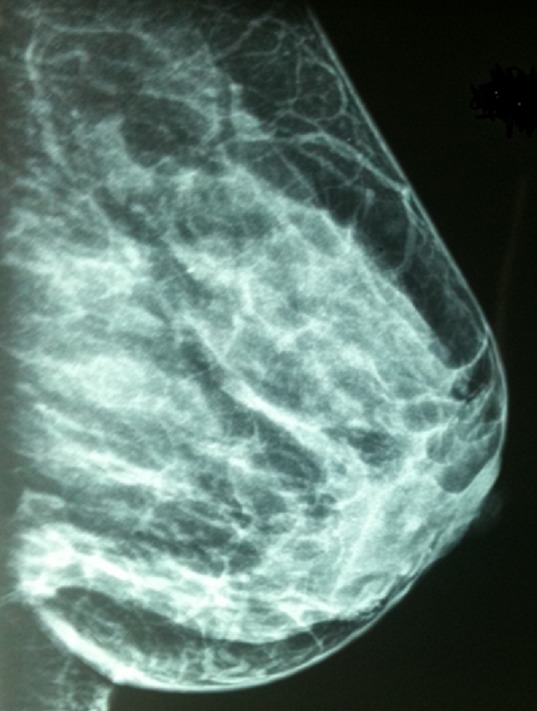
Mammographie de profil de Madame BL montrant une opacité de la jonction des quadrants inferieurs classée ACR5

Madame BL, 50ans, ménopausée, sans antécédent pathologique particulier, a consulté pour tuméfaction du sein droit apparue depuis 2ans ayant augmenté progressivement de volume, l'examen a objectivé un nodule du sillon sous mammaire 4/3cm, dure mal limité, adhérent aux deux plans. Le sein controlatéral, les aires ganglionnaires et le reste de l'examen étaient sans particularités. Une mammographie – échographie mammaire (Figure 3, Figure 4): lésion tissulaire hypoéchogène hétérogène de la jonction des quadrants inferieurs (24mm) classée ACR5. Une microbiopsie au tru-cut a été en faveur d'un carcinome épidermoïde. Un bilan d'extension (échographie abdomino pelvienne, radio pulmonaire) est revenu normal. Une mastectomie totale avec curage ganglionnaire a été réalisée vu la non acceptation par la patiente d'un éventuel traitement conservateur et a confirmé la présence d'un carcinome épidermoïde peu différencié grade III SBR, 4MSBR mesurant 3cm sans extension ganglionnaire sur le curage (12N-/12). Les récepteurs hormonaux positifs (RE=90%, RP=80%), L'Hercept test négatif (score 0); La patiente a été adressée ensuite au service d'oncologie pour traitement adjuvant.

## Discussion

Les carcinomes épidermoïdes primitifs du sein sont rares avec une fréquence de 0,1 à 2% de l'ensemble des carcinomes du sein; ils appartiennent au groupe hétérogène des carcinomes métaplasiques mammaires [1].

L'histogénèse de cette entité est encore controversée [2]: la cellule d'origine ayant subi la métaplasie n'est pas encore déterminée: cellules épithéliales, myoépithéliales ou totipotentes de réserve [3]. Certains auteurs incriminent l'inflammation chronique constatée dans les abcès, mastites chroniques et les sites de biopsies dans la genèse de ces tumeurs. La métaplasie squameuse bénigne du revêtement pourrait en être un précurseur potentiel [4].

Le carcinome épidermoïde du sein touche la femme entre 30–80ans avec prédominance à l’âge de 55 ans. Il touche aussi bien le sein droit que le sein gauche. L'atteinte bilatérale étant rare. Il n'ya pas de présentation clinique ou mammographique particulière: La taille de la tumeur est variable selon le stade de l’évolution [5, 6]. Cependant, l’échographie mammaire soulève l'importance de la nécrose ainsi que l'aspect kystisé des lésions, ce qui suggère que le diagnostic différentiel d'un kyste mammaire complexe pourrait se poser avec le carcinome épidermoïde primitif du sein. Nous n'avons pas observé de formes kystisées comme cela a été décrit dans la littérature. Le diagnostic peut être réalisé par une simple biopsie comme dans nos cas, mais l’étude histopathologique reste indispensable [7].

Histologiquement, le carcinome épidermoïde a la même architecture et les caractères cytonucléaires que son similaire qui se développe dans un autre site. Cependant, le diagnostic ne peut être retenu que lorsque certaines conditions sont remplies: éliminer la possibilité d'une extension locale d'un carcinome épidermoïde du revêtement cutané en regard ou du mamelon et d'une métastase à partir d'un carcinome épidermoïde à distance [8].

L'immunohistochimie montre une expression des cellules tumorales épithéliales des cytokératines de haut poids moléculaire et l'absence d'expression des marqueurs endothéliaux vasculaires. La majorité de ces tumeurs n'expriment pas les récepteurs hormonaux, chez nos patientes: les RHet l'Hercept test étaient négatifs dans 2/3 cas [8].

Ce type de tumeur a la réputation d’être peu lymphophile et dans 70% des cas le curage est dépourvu de métastase ganglionnaire [2]. Sur les 3 cas que nous rapportons, une seule patiente avait un ganglion métastatique. Les études moléculaires démontrent une hyperexpression de la protéine p53 et des marqueurs de l'angiogenèse VEGF et HIF-1α [9]. Le traitement du carcinome épidermoïde du sein est similaire à celui du carcinome canalaire infiltrant. La chirurgie conservatrice est possible; Pour les tumeurs plus grandes, la mastectomie avec curage ganglionnaire est indiquée suivie d'une radiothérapie et d'une chimiothérapie. L'utilisation de l'hormonothérapie est limitée par l'absence de l'hormonodépendance de ce cancer[3, 10].

En situation néoadjuvante, certains auteurs ont rapporté une réponse significative avec régression de la taille tumorale et des signes inflammatoires pour des tumeurs localement avançées en utilisant le 5 Fluorouracile et le cisplatine[3]. Sur les 2 cas que nous rapportons et qui ont reçu une chimiothérapie en néoadjuvant, dont un à base de cisplatine la réponse thérapeutique n’était pas optimale.

La taille tumorale et l'envahissement ganglionnaire axillaire sont retenus comme étant les principaux facteurs pronostiques dans la littérature [2]. Certains auteurs considèrent que les facteurs prédictifs d'une mauvaise évolution des cancers épidermoïdes sont la composante fusiforme, la nécrose, et l'acantholyse cellulaire [1].

Le pronostic des carcinomes épidermoïdes reste péjoratif, le siège de prédilection des métastases survenant au cours des cinq premières années est le poumon, le foie, l'os ou le cerveau. La survie moyenne à 5ans est estimée entre 50 et 63% [2, 3].

## Conclusion

Les carcinomes épidermoïdes du sein sont rares. La présentation clinique et mammographique n'est pas spécifique, l'aspect kystisé des lésions et la présence de nécrose sont recherchés à l’échographie mammaire. Le diagnostic est histologique. Ce cancer est réputé être peu lymphophile et non-hormonodépendant. Le traitement rejoint celui des carcinomes infiltrant canalaires et repose sur la chirurgie, la radiothérapie et la chimiothérapie. Le pronostic reste péjoratif ce qui impose l’étude de séries plus larges permettant de mieux connaitre leur histogénèse et de prévoir leur profil évolutif afin de mieux codifier sa prise en charge.
